# Forging Neuroimaging Targets for Recovery in Opioid Use Disorder

**DOI:** 10.3389/fpsyt.2019.00117

**Published:** 2019-03-07

**Authors:** Jennifer L. Stewart, April C. May, Robin L. Aupperle, Jerzy Bodurka

**Affiliations:** ^1^Laureate Institute for Brain Research, Tulsa, OK, United States; ^2^Department of Community Medicine, University of Tulsa, Tulsa, OK, United States; ^3^Joint Doctoral Program in Clinical Psychology, San Diego State University, University of California, San Diego, San Diego, CA, United States

**Keywords:** opioid use disorder, neuroimaging, magnetic resonance imaging, electroencephalography, event related potentials, recovery, abstinence

## Abstract

The United States is in the midst of an opioid epidemic and lacks a range of successful interventions to reduce this public health burden. Many individuals with opioid use disorder (OUD) consume drugs to relieve physical and/or emotional pain, a pattern that may increasingly result in death. The field of addiction research lacks a comprehensive understanding of physiological and neural mechanisms instantiating this cycle of *Negative Reinforcement* in OUD, resulting in limited interventions that successfully promote abstinence and recovery. Given the urgency of the opioid crisis, the present review highlights faulty brain circuitry and processes associated with OUD within the context of the *Three-Stage Model of Addiction* (1). This model underscores *Negative Reinforcement* processes as crucial to the maintenance and exacerbation of chronic substance use together with *Binge/Intoxication* and *Preoccupation/Anticipation* processes. This review focuses on cross-sectional as well as longitudinal studies of relapse and treatment outcome that employ magnetic resonance imaging (MRI), functional near-infrared spectroscopy (fNIRs), brain stimulation methods, and/or electroencephalography (EEG) explored in frequency and time domains (the latter measured by event-related potentials, or ERPs). We discuss strengths and limitations of this neuroimaging work with respect to study design and individual differences that may influence interpretation of findings (e.g., opioid use chronicity/recency, comorbid symptoms, and biological sex). Lastly, we translate gaps in the OUD literature, particularly with respect to *Negative Reinforcement* processes, into future research directions involving operant and classical conditioning involving aversion/stress. Overall, opioid-related stimuli may lessen their hold on frontocingulate mechanisms implicated in *Preoccupation/Anticipation* as a function of prolonged abstinence and that degree of frontocingulate impairment may predict treatment outcome. In addition, longitudinal studies suggest that brain stimulation/drug treatments and prolonged abstinence can change brain responses during *Negative Reinforcement* and *Preoccupation/Anticipation* to reduce salience of drug cues, which may attenuate further craving and relapse. Incorporating this neuroscience-derived knowledge with the *Three-Stage Model of Addiction* may offer a useful plan for delineating specific neurobiological targets for OUD treatment.

## The Devastation of Opioid Use Disorder

Opioid use disorder (OUD) is a chronic, relapsing condition, associated with a staggering $75 billion public health burden and millions of years of premature mortality, attributable to a 350% increase in opioid-related deaths over the past two decades (2, 3). In 2016, more than 60 million patients had used and misused opioid-based anti-pain medication despite growing awareness of negative consequences and reduced effectiveness of long-term use (4). It is estimated that 20–30% of opioid-related overdoses are actually intentional suicide attempts, as opposed to accidents (5). It is not surprising that OUD-related suicide risk is over six times the national average, as individuals with OUD are struggling with disproportionate amounts of aversive mood states (anhedonia, dysphoria, suicidal ideation, irritability, anger, guilt, and shame) that are associated with heightened stress and drug craving (5–10). Moreover, the longer the temporary abstinence from drug use, the greater attention users devote to bodily sensations signaling a homeostatic imbalance. The process of attending to these sensations in an attempt to restore homeostasis, also known as allostasis (11), contributes to increased craving and withdrawal (9). Users actively attempt to avoid withdrawal comprised of agonizing physiological states (e.g., sweating, racing heartbeat, fever, nausea/vomiting, stomach cramps, diarrhea, generalized pain, depression, and anxiety) starting within hours of last use and lasting for days (12, 13). Opioid consumption relieves symptoms of negative affect as well as craving/urges in individuals with OUD (14), thereby increasing the likelihood of future drug use in the presence of negative affective and physical states, a process known as negative reinforcement. In short, individuals with OUD consume drugs to relieve emotional and/or physical pain. A *Three-Stage Model of Addiction* based on substantial animal and human studies highlights the importance of negative reinforcement, as well as binging and anticipation processes, to the exacerbation and maintenance of chronic substance use (1, 15). This model can be applied to various substance use disorders and further expanded to elucidate processes unfolding as a function of prolonged abstinence from use. At this point in time, however, we lack a comprehensive understanding of the underlying physiological and neural mechanisms involved in allostasis and negative reinforcement processes. As a result, we possess limited interventions to promote recovery and abstinence, and are left treating symptoms rather than underlying biological systems contributing to OUD.

Successful overdose-reversal and OUD treatment interventions are urgently needed to reduce mortality, increase quality of life, and lessen economic burden to society and healthcare systems. Modern neuroimaging technology advanced our ability to measure and quantify structural abnormalities and disrupted functionalities of brain circuitry. Neuroimaging research can be particularly beneficial for identifying brain circuitry and systems underlying allostasis and aversive states within OUD, thus leading to identification of targets for pharmacological and behavioral interventions to aid in addiction recovery. The goals of the present review are to: (1) highlight faulty brain circuitry and processes associated with OUD within the context of a *Three-Stage Model of Addiction* (1, 15); (2) discuss strengths and limitations of this imaging work with respect to study design and when available, individual differences such as opioid use chronicity/recency, comorbid symptoms, and biological sex that may influence interpretation of findings; and (3) translate gaps in the OUD literature into future research directions to lead toward a neuroscience-informed understanding of individual differences and potential points for intervention.

## Framing OUD Research Within the Neurocircuitry of Addiction

**Figure 1:**
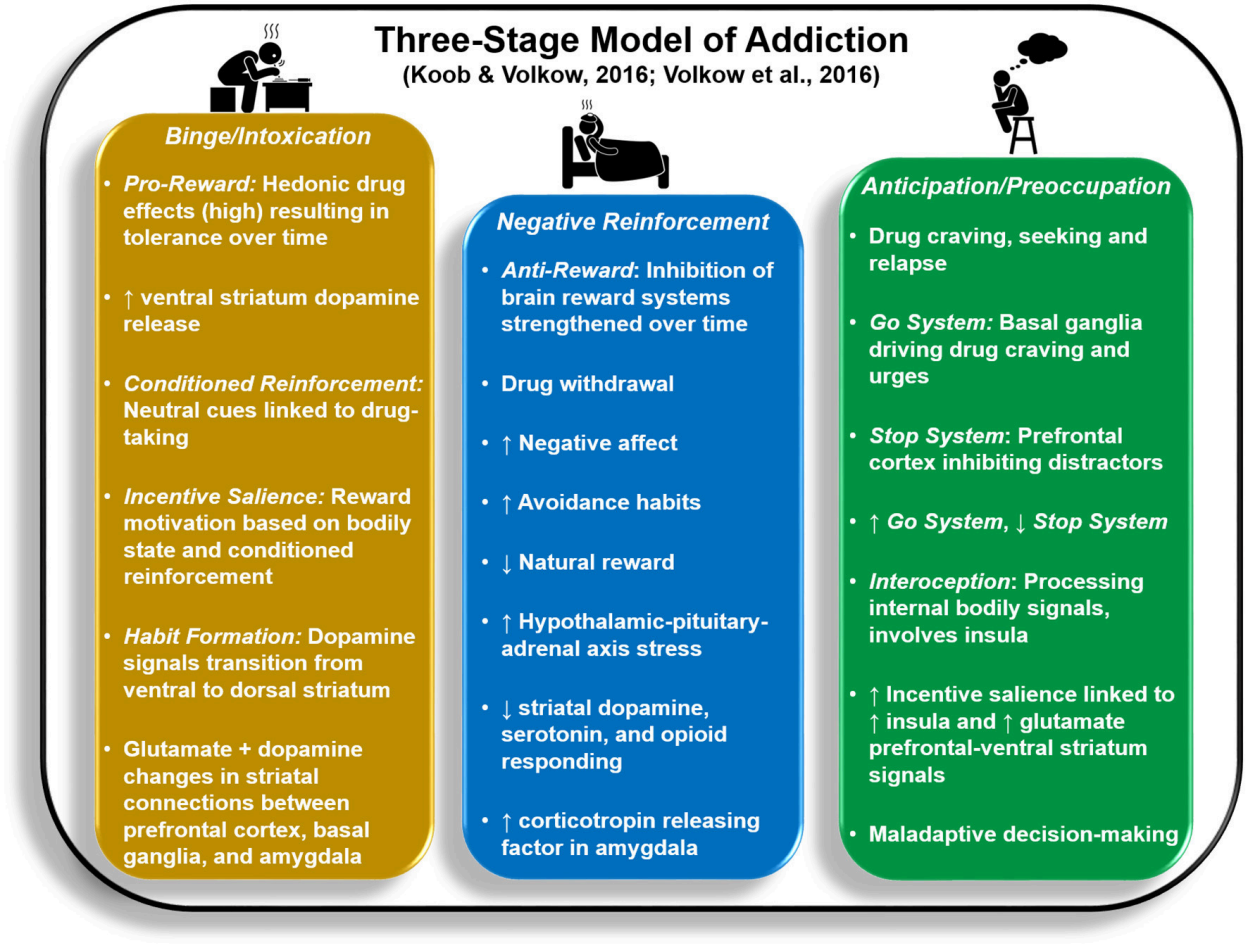
Key psychological and neurobiological processes reflected in the *Three-Stage Model of Addiction* (1, 15).

It is argued that three stages of motivational dysregulation instantiate and maintain the chronic cycle or stages of addiction: *Binge/Intoxication, Negative Reinforcement*, and *Preoccupation/Anticipation* (1, 15, 16). Within this model, these stages, which are likely not entirely separable from each other, are linked to aberrant patterns of activity within/between brain regions involved in reward processing [ventral striatum (VS)], cognitive control [frontocingulate regions including inferior frontal gyrus (IFG) and anterior cingulate cortex (ACC)], aversive emotional states [amygdala (AMG)], and a sense of the internal body state, known as interoception [insula (INS)]. Figure 1 illustrates psychological and neurobiological processes associated with each stage.

Whereas the *Binge/Intoxication* stage lays the groundwork for initial transition to addiction, the latter two stages act to drive drug relapse. *Binge/Intoxication* reflects positive reinforcement processes that begin with recreational drug use, wherein rewarding consequences of drug use (e.g., euphoria, high), accompanied by increased VS (nucleus accumbens, globus pallidus) activity and dopamine release, increase the likelihood of future drug consumption. This cycle eventually leads to impulsive, intensified use that is difficult to control. Both animal and human research demonstrate that *Binge/Intoxication* initially weakens the brain's response to natural rewards while increasing drug tolerance by remapping striatal circuitry (consisting of decreased VS activity paired with increased dorsal striatum responses) to prioritize habitual drug rewards, a process termed incentive sensitization (17–20).

The *Negative Reinforcement* stage is thought to strengthen the likelihood of future drug use by reducing aversive mood, stress, and withdrawal states exacerbated by lack of recent drug administration. It is argued that a compulsive, habitual cycle persists: heightened anxiety and stress are briefly reduced as a result of drug use, but then build up over time, leading to obsessions about future drug-taking until the drug is used again (21). The extended AMG (comprised of AMG central nucleus, bed nucleus of the stria terminalis, and posterior nucleus accumbens shell) interacts with hypothalamic regions involved in neurochemical stress reactions and is also linked to aversive emotional reactions in humans (21). The stria terminalis, in particular, is implicated in norepinephrine hyperactivity associated with opioid withdrawal (22). Researchers theorize that stress-related brain systems/circuitry are activated first during the *Binge/Intoxication* stage to counteract excessive dopamine release; over time, neurochemical stress signals are thought to suppress dopaminergic responsivity to drug reward (23).

It is argued that the *Preoccupation/Anticipation* stage involves obsessive thoughts about future drug-taking that are prioritized over other goals, paired with weakened inhibitory control over drug craving/urges (1). Substantial evidence implicates INS in drug craving and aversive feeling states linked to withdrawal and short-term abstinence (24–26). In addition, heightened prefrontal cortex (PFC) and ACC activities evident within the context of drug cue-elicited craving theoretically drive increased preoccupation with and motivated actions toward drug-taking (25). While drug cues are often associated with exaggerated INS, ACC, and PFC responses (27), decision-making involving non-drug stimuli reflects attenuation in these regions as a function of addiction (28–30). With respect to recovery from drug addiction, however, it is still unclear how brain mechanisms implicated in *Preoccupation/Anticipation* and *Negative Reinforcement* stages change as a function of detoxification, early abstinence (e.g., 1–3 months sober), and prolonged abstinence (e.g., greater than 1 year sober), particularly within the same individuals over time, and whether brain changes parallel reductions in wanting to use drugs. As we review neuroimaging studies below, whenever possible we couch findings within the context of participant abstinence duration to develop predictions for what functions might improve with sobriety.

Taken together, neuroimaging studies provide compelling evidence that striatal, frontocingulate, AMG, and/or INS structure, function, and/or connections are disrupted in OUD. What do these disruptions mean with respect to specific impairments in OUD? Research findings indicate that the meaning of INS dysfunction depends on the particular location that is affected. Anterior INS, connected to IFG and dorsal striatum, is implicated in awareness of bodily feeling states as well as the learning and implementation of goal-directed actions that can be conceptually linked to cognitive control processes, whereas ventral INS is more strongly connected to AMG and VS and is thought to be involved in emotional salience and affective feeling states. In contrast, middle and posterior INS are connected with somatosensory regions (sensory and parietal cortices) associated with the processing of bodily feeling states, including pain signals (31, 32). Dorsolateral PFC is thought to work with ACC to regulate goal-directed behavior, wherein it is argued that dorsal ACC processes the value and difficulty of behavior change via its connections with dorsolateral PFC as well as AMG, dorsal striatum, and primary motor cortex (33). Within the context of stress, cognitive control functions in frontocingulate and anterior INS regions are argued to be hijacked by AMG connections. For example, although the dorsolateral PFC is thought to play an active role in pain suppression (34), within the context of aversive events, heightened AMG signals activate neurochemical stress reactions that serve to downregulate dorsolateral PFC in favor of salience-driven habitual, impulsive responses instantiated via dorsal striatum (35). Moreover, greater functional and structural links between basolateral AMG and anterior INS are associated with higher state and trait anxiety (36), instantiating aversive feeling states accompanying stress.

**Figure 2:**
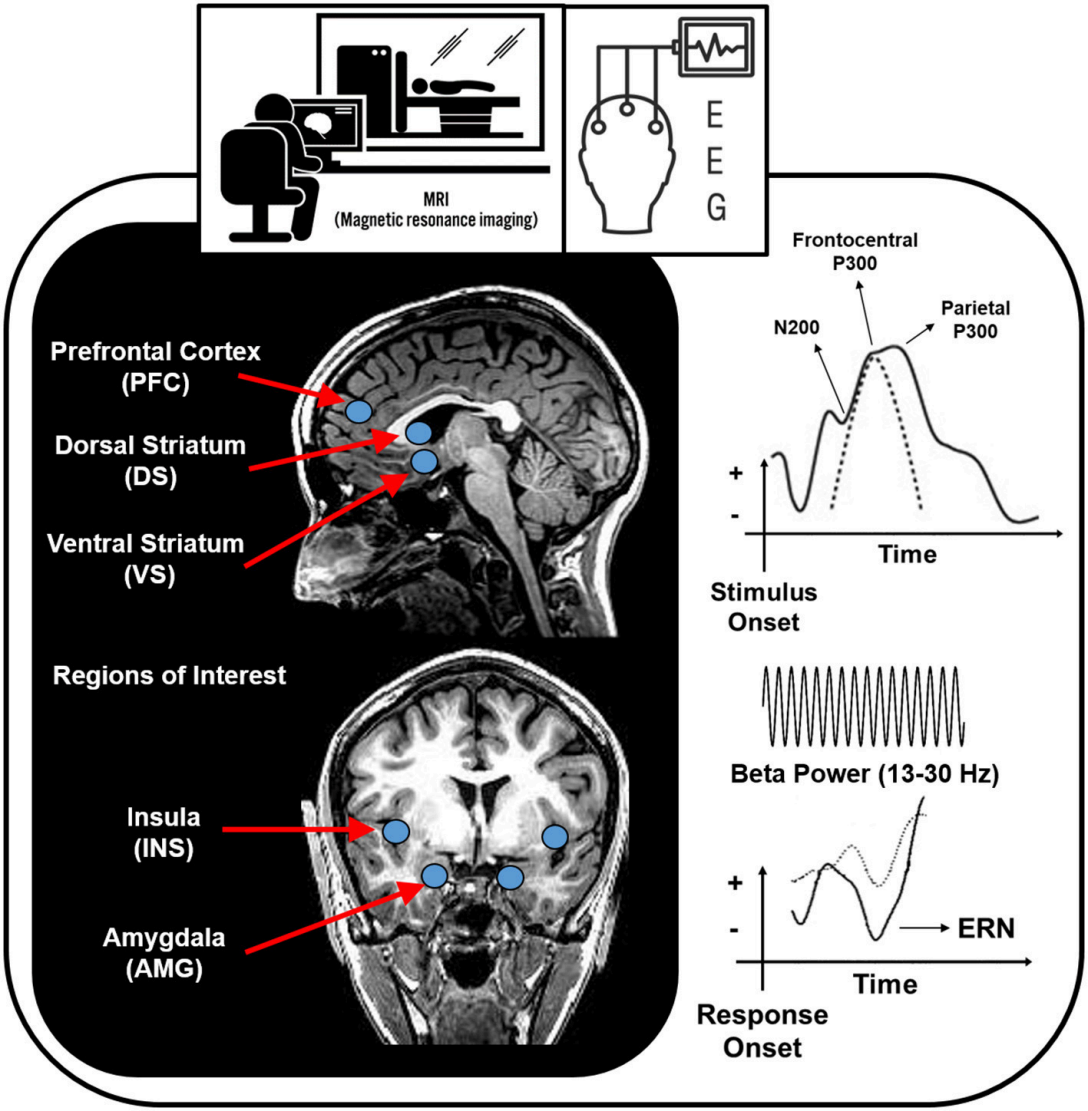
Brain regions and processes that potentially map onto *Negative Reinforcement* and *Anticipation/Preoccupation* stages of the *Three-Stage Model of Addiction* (1, 15). EEG, electroencephalography; ERN, error related negativity.

**Figure 3:**
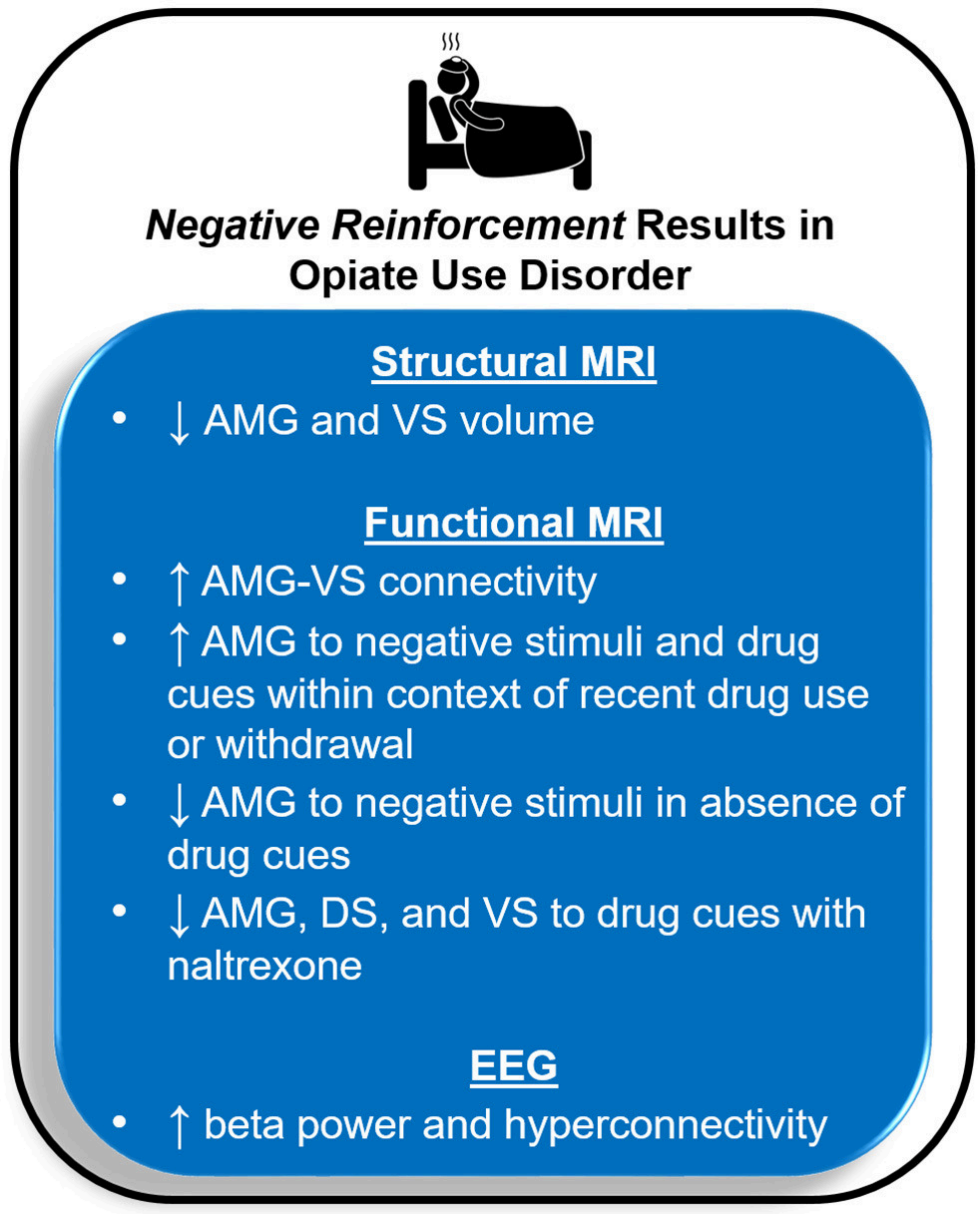
Magnetic resonance imaging (MRI) and electroencephalography (EEG) results for opioid use disorder that may map onto the *Negative Reinforcement* stage of the *Three-Stage Model of Addiction* (1, 15). AMG, amygdala; VS, ventral striatum; DS, dorsal striatum.

**Figure 4:**
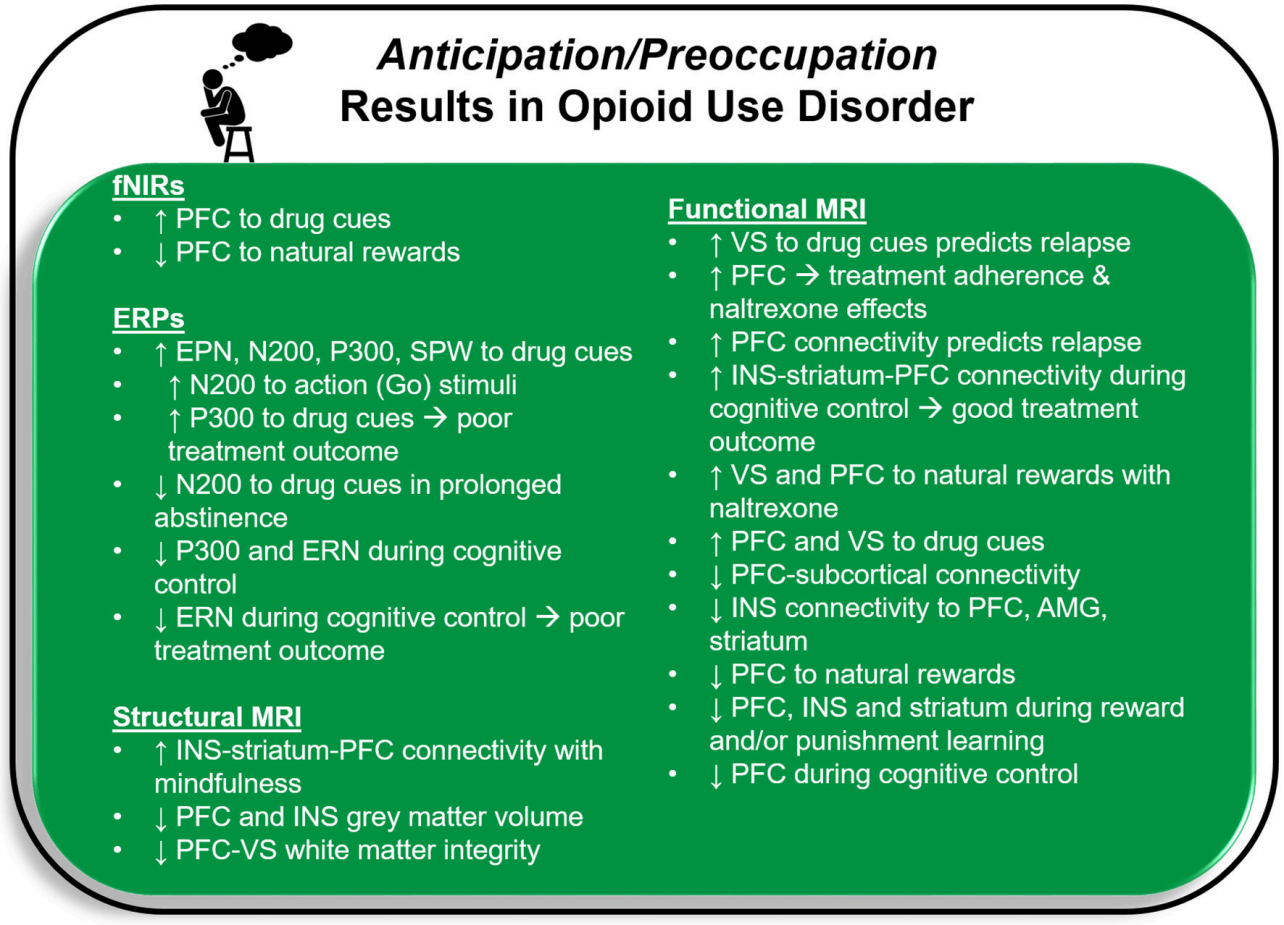
Functional near-infrared spectroscopy (fNIRs), event related potential (ERP), and magnetic resonance imaging (MRI) results for opioid use disorder that may map onto the *Anticipation/Preoccupation* stage of the *Three-Stage Model of Addiction* (1, 15). PFC, prefrontal cortex, including anterior cingulate cortex; EPN, early positive negativity; SPW, slow positive wave; ERN, error related negativity; INS, insula; VS, ventral striatum; AMG, amygdala.

Deficits in the brain circuitry outlined above are present in conjunction with aberrant timing and allocation of neural resources to drug and non-drug related stimuli, consistent with the *Three-Stage Model of Addiction*. In the following sections, specific neuroimaging tools related to magnetic resonance imaging (MRI), functional near-infrared spectroscopy (fNIRs), electroencephalography (EEG), event related potentials (ERP), repetitive transcranial magnetic stimulation (rTMS) and deep brain stimulation (DBS) are briefly explained and cross-sectional and longitudinal OUD-relevant literature is summarized for each technique. Figure 2 illustrates brain regions and processes of interest that are described in more detail below. Next, Figures 3 and 4 summarize brain findings that appear to map onto *Negative Reinforcement* and *Anticipation/Preoccupation* stages. To compile research articles for this review, combinations of the following search terms were entered in Google Scholar: “opioid,” “heroin,” “MRI,” “EEG,” “rTMS,” “fNIRs,” “DBS,” “ERP,” “prescription opiate,” “methadone,” “naltrexone,” “therapy,” “abstinence,” “relapse,” “resting state fMRI,” and “buprenorphine.”

### Structural MRI (sMRI)

With its high spatial resolution (typically in order of 1 mm3), sMRI offers ways to differentiate different brain tissues, such as gray and white matter, and to quantify gray and white matter volume within various brain regions. Gray matter consists of cell bodies, dendrites, unmyelinated axons, and synapses that facilitate specialized information processing in cortical and subcortical regions, whereas white matter consists of myelinated axons that relay signals from one brain region to another. Studies employing sMRI demonstrate that OUD is characterized by attenuated gray matter volume and white matter integrity in/surrounding striatum, frontocingulate regions (including IFG), AMG, and INS, with higher opioid use chronicity, use recency, and depression symptoms linked to greater reductions in specific regions (37–41). For instance, greater opioid use chronicity is associated with lower frontocingulate and/or INS cortical thickness in active as well as abstinent OUD users (37, 42, 43) in addition to decreased VS gray matter volume (44). Moreover, within individuals on opioid maintenance treatment for OUD, lower VS volume is associated with higher depression symptoms, whereas lower AMG volume is linked to greater daily opioid dose (40). Gray matter reductions within orbito-medial prefrontal cortex and bilateral globus pallidus are also associated with increased cognitive impulsivity among individuals on opioid maintenance treatment (45). With respect to abstinence, higher compulsive behavior reported by sober individuals with OUD is linked to lower white matter surrounding VS and rostral ACC when compared to that of active OUD users and healthy controls (44). In summary, brain regions implicated in *Binge/Intoxication* (VS), *Negative Reinforcement* (AMG), and *Preoccupation/Anticipation* stages (PFC, ACC, and INS) show structural attenuations, ostensibly contributing to various information processing impairments that may have a stronger impact when users are attempting to resist using opioids. For instance, VS attenuation may reflect the capacity for heightened drug tolerance and reduced euphoric effects of drug consumption. Additionally, PFC, ACC, and anterior INS volume reductions could manifest in impairments in adaptive goal-directed behavior, whereas diminished AMG structure might manifest in dysregulated stress and salience signaling in the presence/absence of drugs.

### Functional MRI (fMRI)

fMRI offers good spatial resolution (typically in order of a few mm3) to detect and measure temporal changes in blood flow, volume, and blood oxygenation (e.g., blood oxygenation level dependent, or BOLD contrast) while individuals are resting or performing various tasks. Active neurons in the brain require oxygenated blood to replenish energy; BOLD fMRI is affected by the differences in magnetic susceptibility between deoxygenated and oxygenated blood, and by local increases in blood flow and volume, signaling brain regions that are more active during one particular condition, stimulus, response, or timeframe vs. another. Researchers often quantify brain changes by computing the percent signal change between an active condition and a baseline condition. It is argued that the characterization of spontaneous (or intrinsic) brain signals during a resting state (e.g., without any particular task involved) are just as worthy of study as brain signals evoked by a particular stimulus and/or response because these spontaneous measurements reflect degree of energy consumption required to maintain default functioning in the absence of particular task demands (46, 47). Most fMRI research in OUD focuses on either drug-cue valuation processes compared to neutral cues and/or natural rewards (food, sex, social interactions, money), or decision-making in the absence of emotional, reward, or drug-related cues. Only a few studies have examined brain mechanisms involved in responses to negative stimuli, limiting interpretability.

#### Resting-State fMRI

Studies of spontaneous fMRI often focus on coherence (or connectivity) of signals across multiple spatially distinct cortical and subcortical brain regions. OUD is associated with weak frontocingulate functional connectivity with subcortical regions, but strong functional connectivity within subcortical regions such as striatum and AMG (48), findings consistent with a reward-control imbalance in OUD [stronger reward-stress connectivity paired with weaker cognitive control connectivity; (49)]. Multiple fMRI studies report weakened INS connectivity to IFG, striatum, and AMG, with those testing positive for opioids or reporting greater opioid use chronicity exhibiting the greatest dysfunction, findings in line with the *Preoccupation/Anticipation* stage (41, 49, 50). Finally, research indicates that individuals with OUD exhibit attenuated ACC activity and reduced connectivity with PFC and striatal regions; moreover, lower ACC signal within this context is linked to greater drug cue-induced craving (51, 52).

#### Task-Based fMRI: Cue Reactivity and Non-Drug Rewards

OUD is marked by frontocingulate and striatal hyperactivation to drug cues, particularly within active users (up to a few hours sober), with degree of response decreasing as a function of longer abstinence (i.e., 6–14 months as opposed to 1 month), findings consistent with the *Preoccupation/Anticipation* stage of addiction (53–62). Compared to non-substance using individuals, those with OUD show frontal attenuation to pleasant non-drug stimuli such as food, pornography, and interactive social situations (54, 63), although this pattern may dissipate as a function of abstinence [3 years; (54)]. With respect to reward sensitivity, users with OUD exhibit difficulty distinguishing between non-drug win and no-win outcomes in striatal brain regions (64); moreover, individuals with OUD show INS, ACC, and IFG attenuation during win/loss anticipation and feedback (65) in line with the *Preoccupation/Anticipation* stage of addiction.

#### Task-Based fMRI: Cognitive Control

OUD is associated with frontocingulate hypoactivation during tasks requiring sustained attention, working memory, and/or cognitive/behavioral inhibition compatible with the *Anticipation/Preoccupation* stage of addiction, with fMRI studies reporting this pattern regardless of abstinence duration or presence of opioid-replacement treatment (66–69). One study demonstrates no difference in ACC activation between users with OUD on opioid replacement therapy (buprenorphine or methadone) and non-users during behavioral control. However, users do not show a positive correlation between ACC activation and behavioral performance as seen in non-users, indicating a notable discrepancy between brain signaling and behavior (70); these findings suggest that even when recruited, these regions may not function as effectively for OUD. Some evidence suggests that cognitive control functions involving IFG and ACC may improve as a function of prolonged abstinence in OUD, given that former opioid users abstinent for at least 6 months perform similarly to healthy individuals and/or better than users on opioid replacement therapy during cognitive control tasks. However, the literature is far from conclusive and mixed results may be due, in part, to variability in opiate use chronicity and recency across studies (39).

#### Task-Based fMRI: Aversive Stimuli

On the whole, very limited research suggests that OUD is characterized by blunted brain responses to negatively valenced stimuli as well as punishing outcomes in the absence of drug cues. Two fMRI studies report hypoactive AMG responses to negative and positive as opposed to neutral stimuli in OUD individuals who are abstinent 2–5 months (71) as well as current users with OUD; it is important to note that these results are based on samples with comorbid borderline personality disorder who are also on opioid replacement therapy (72). Thus, findings may not easily generalize to other OUD samples. These reports of blunted AMG signals are the opposite of what would be predicted by the *Negative Reinforcement* stage, which suggests that AMG responses should be intensified as a function of aversive cues. In contrast, two fMRI studies demonstrate that drug cues evoke AMG hyperactivation in individuals with OUD who are expecting to consume opioids or have recently withdrawn from opioids, potentially reflecting exaggerated salience associated with drug cues and/or bodily signals that in the past have signaled opioid withdrawal. More specifically, when active OUD users are administered saline as opposed to opioids, they display greater AMG activation than healthy individuals to fearful faces, a pattern that is linked to elevated state anxiety (73). Similarly, newly detoxified individuals with OUD exhibit hyperactive AMG responses to drug as opposed to neutral films, a pattern correlated with heightened craving (74). Furthermore, OUD patients on methadone replacement exhibit greater INS and AMG activation to opioid cues before as opposed to after ingestion of their daily methadone dose (75). Drug cues in abstinent individuals with OUD also appear to act as salient stimuli, linked to heightened anxiety, other negative emotions, and physiological blood pressure/heart rate increases (76). On the whole, these findings are accordant with the *Negative Reinforcement* stage.

Non-imaging data indicate that active OUD is associated with exaggerated self-reported arousal to negative non-drug images (77), suggesting that additional brain-behavior research is needed to determine whether patterns of AMG response to emotional stimuli change as a function of abstinence. Greater negative affect induced by film clips still increases drug craving in OUD users without the presence of drug cues, congruent with the *Negative Reinforcement* stage of addiction; furthermore, this relationship is stronger for users with high as opposed to low anxiety sensitivity (78). Moderation by anxiety sensitivity points to the importance of measuring individual differences in users' perceptions and awareness of bodily sensations, as these may intensify stress responses that hijack abstinence efforts.

Lastly, OUD is linked to difficulty differentiating punishing vs. non-punishing feedback within striatum (64). Behavioral studies indicate that individuals with active and/or former OUD show difficulties avoiding punishment (79–81) and demonstrate heightened risk-taking following punishment (82). This pattern of impaired decision-making in the face of punishment may be more relevant to the *Preoccupation/Anticipation* than the *Negative Reinforcement* stage, as a meta-analysis implicates INS in the implementation of punishment-related prediction errors and ACC and PFC regions in reinforcement-based decision making more generally (83).

### Functional Near-Infrared Spectroscopy (fNIRs)

The fNIRs technology employs near-infrared light attenuation to quantify concentration of oxy- and deoxy-hemoglobin. fNIRs can differentiate skin, skull, and cortical surface tissue, and produce a BOLD contrast similar to fMRI, however without the ability to measure whole brain responses. Studies using this technology indicate that OUD patients recently detoxified from opioids show: (1) greater right dorsolateral PFC activation to opioid cues than individuals with OUD abstinent for at least 2 months (84); and (2) higher anhedonia symptoms paired with lower rostral and/or ventrolateral PFC to appetitive food and positive social interactions than healthy individuals (63). These results point to greater attentional resources being devoted to drug cues than other types of rewards, consistent with the *Preoccupation/Anticipation* stage of addiction.

### Electroencephalography (EEG)

#### EEG Time and Frequency Domains

EEG, the continuous recording of ongoing brain electrical activity via scalp electrodes, possesses high temporal resolution (order of milliseconds) (85). Resting state EEG recordings measure the brain's pseudo-periodic oscillatory activity due to coherent activity from many neurons synchronized in time and space. For EEG signal frequency analyses, a Fast Fourier Transform (FFT) technique decomposes the EEG time series into a frequency spectrum by voltage (a measure of signal magnitude, or amplitude) matrix; this information can then be segmented as a function of specific frequency “bands” that are associated with various mental processes. Frequencies most studied in OUD samples include those segmented within theta, alpha, beta, and gamma bands. Theta band (4–7 Hz) activity is implicated in cognitive control processes including working memory and error monitoring (86–88). Decreases in alpha band (8–13 Hz) activity are associated with increases in active information processing involving attention (89), whereas beta band (13–30 Hz) decreases signal an impending voluntary motor action (90). Finally, gamma band (30–100 Hz) activity is theorized to reflect the comparison of a stimulus with information held in memory to determine a match or mismatch (91). EEG power (the square of the EEG magnitude of the signal amplitude within a particular band) is often calculated to compare between clinical groups or conditions. In addition, EEG coherence metrics are calculated to reflect how strongly oscillations between two or more measuring electrodes reflecting and mapping into synchronized brain regions activities within a particular frequency band.

Although EEG frequencies can be measured within the context of a particular task, resting-state EEG studies investigating frequency band differences as a function of OUD are the norm. On the whole, this literature indicates that EEG power and coherence are disrupted in chronic OUD users compared to healthy individuals, although findings are inconsistent as to directionality (which group is higher or lower) as well as which frequency band, hemisphere, or specific brain region is affected and whether these patterns normalize as a function of abstinence or methadone maintenance (92, 93). However, EEG frequency studies of OUD are atheoretical with respect to how findings map onto stages of addiction or cognitive/emotional functioning, and low spatial resolution of most EEG recording montages limit spatial (brain) localization of frequency signals within OUD samples.

The most consistent finding is that individuals with OUD (whether actively using, maintained on methadone for at least 6 months, or in the early stages of abstinence) exhibit greater beta power than healthy individuals [91–93). With respect to longer abstinence duration, one study reports no difference in beta power between healthy controls and OUD users abstinent 1–6 months, whereas another study indicates that beta power decreases as a function of longer OUD abstinence (94). As beta power increases are thought to reflect decreased need for future motor actions, these results suggest that active opioid users can be characterized by reduced behavioral activation, at least during intrinsic processing. Additional research probing beta power changes during reward and stress states in opioid users may contribute to our understanding of *Binge/Intoxication, Negative Reinforcement*, and *Preoccupation/Anticipation* stages within the context of OUD. Perhaps beta power changes as a function of prolonged abstinence can track stages of recovery, although longitudinal studies are warranted to test this hypothesis.

In contrast to beta band results, findings for the alpha band are somewhat mixed, with: (1) active OUD users exhibiting either higher (93) or lower (95) power than healthy comparison subjects; (2) OUD users maintained on methadone for 6+ months displaying lower (96) or higher (93) power than non-users; and (3) abstinent OUD users showing similar levels of power as healthy individuals (97) or increasing alpha power as a function of sobriety duration (94). For theta band activity, active OUD users either exhibit lower (95) or higher (93) power than healthy individuals. However, OUD users abstinent 1–6 months display similar theta power as control subjects (97), findings suggestive of a state-like change in theta power as a function of current drug use. Time frequency analysis of short duration EEG frequency band distribution (as opposed to averaging frequency bands across the entire length of EEG recording) indicate that active OUD users exhibit higher occurrence of alpha and beta rhythms but lower occurrence of theta rhythms than comparison subjects; moreover, OUD users show greater occurrence of these rhythms in the right than the left hemisphere (98); these findings could be consistent with fMRI data suggesting weakened right frontal processing in OUD that could reflect inhibitory impairments associated with faulty IFG/ACC signaling, consistent with the *Preoccupation/Anticipation* stage of addiction.

With regard to EEG coherence within and across regions of the brain, active OUD exhibit local hyperconnectivity in alpha and beta frequency bands, a pattern that does not change as a function of early (2-week) abstinence. However, remote alpha and beta hypoconnectivity evident in active OUD users does appear to normalize during the early stages of sobriety (99, 100). Finally, gamma band findings indicate that active OUD as well as OUD on prolonged methadone treatment display greater gamma power than healthy individuals (50), and OUD abstinent at least 2 weeks exhibit greater fronto-occipital gamma band coherence within the left hemisphere than CTL, although the significance of this greater coherence is not well-understood (101).

#### EEG Event Related Potentials (ERPs)

ERPs are averaged periods of EEG recordings interpreted within the time domain that are elicited by a particular stimulus or a response. ERPs allow researchers to understand the onset and/or duration of perceptual, attentive, and other cognitive and emotional processes (85). Unlike fMRI studies suggesting that faulty cognitive control circuitry may normalize as a function of OUD abstinence, ERP studies provide mixed results, suggesting that this may not be the case (95, 102–110), although greater opioid use chronicity does appear to be associated with greater frontocingulate reductions (103). Temporal resolution differences between ERPs (milliseconds) and fMRI (seconds) suggest that aspects of early stimulus evaluation (measured by multiple ERP amplitude/latency components) are still disrupted in OUD at various stages of abstinence accordant with the Preoccupation/Anticipation stage of addiction.

##### ERP components

Details regarding timing and proposed function of various ERP components, including early posterior negativity (EPN), N200, P300, slow positive wave (SPW) and error related negativity (ERN), are provided below within the context of various paradigms, including cognitive control, cue reactivity, working memory, attention and emotion tasks.

##### EPN

The EPN is a positive ERP deflection occurring 200 ms post-stimulus, thought to reflect and associate with early perceptual processing in temporal/occipital brain regions (111). During an emotional Stroop task involving positive, negative, neutral, and opioid images, OUD users abstinent an average of 9 months show larger EPN amplitude to opioid images than healthy participants in the absence of behavioral differences between groups (109). These results indicate that even with prolonged sobriety, perception of drug cues is prioritized.

##### N200

N200 is a negative ERP deflection occurring 200–350 ms after a stimulus, thought to reflect and associate with conflict monitoring processes (112, 113). During a go/nogo task, individuals with OUD (abstinent for 4 months) show larger frontocentral N200 amplitudes to go (action) trials than healthy controls, but groups do not differ on N200 amplitudes to nogo (inhibition) trials (110); findings imply that neural resources are overly devoted to action tendencies, perhaps related to impulsivity. In contrast, however, former OUD and cocaine users display no N200 differences from non-users during response inhibition tasks involving neutral and emotional stimuli (114). OUD users abstinent at least 1 month show greater N200 amplitude to opioid images during a dot probe task than controls (115), in contrast, OUD users abstinent 8–24 months exhibit smaller N200 to opioid images than healthy subjects (108). These results suggest that addicted individuals experience inhibitory difficulties in the presence of drug cues as represented by the *Preoccupation/Anticipation* stage of addiction that may change as a function of prolonged recovery.

##### P300

P300 is a positive ERP deflection occurring 300–600 ms after a stimulus thought to reflect and associate with attention allocation, motivational salience, and/or updating of short-term memory, depending on the paradigm used (85). Among current OUD, findings point to exaggerated salience of opioid cues at the expense of other stimuli, accordant with the *Preoccupation/Anticipation* stage of addiction. Chronic users with OUD display smaller P300 amplitude and longer P300 latency than healthy individuals during digit span and auditory oddball tasks, but larger P300 amplitude to opioid images during a cue reactivity task (95). P300 responsivity has also been examined among substance users with varying lengths of remission. For example, substance users in residential treatment with a history of addiction (cocaine use disorder with/without alcohol use disorder and OUD) exhibit lower P300 amplitude across the entire cortex than healthy individuals to targets during a visual continuous performance test; furthermore, across the three user groups, shorter abstinence is associated with smaller P300 amplitude (102). Similarly, individuals with OUD who are recently detoxified or on opioid replacement therapy exhibit greater P300 amplitude to opioid images than positive, negative, or neutral images, with larger opioid-related P300 amplitude linked to greater self-reported craving; however, OUD subjects do not differ in P300 amplitude from healthy individuals across conditions (116). Moreover, OUD users abstinent for at least 6 months show smaller P300 amplitudes during a working memory task than healthy individuals and current OUD users in frontal regions (105, 106). However, OUD users, their first-degree relatives, and healthy controls do not differ in P300 amplitude to auditory oddball targets (107). Overall, findings among recently abstinent and treatment-seeking individuals are inconsistent as to whether neural resources devoted to attention/salience of non-drug cues improve as a function of abstinence.

##### SPW

The SPW is a positive frontal ERP deflection that onsets at least 600 ms post-stimulus and lasts for several 100 ms, reflecting and associated with sensitivity to emotional valence as well as motivational salience (117, 118). OUD users abstinent for a minimum of 2 weeks show greater SPW amplitude to opioid than neutral images, whereas healthy individuals show no difference between opioid and neutral pictures; moreover, within users, greater central SPW amplitudes are associated with heightened arousal to opioid cues (101). These results are in line with SPN and P300 findings for opioid cues, indicating heightened resources devoted to drug cues in active or early-abstinent users with OUD.

##### ERN

The ERN is a negative ERP deflection occurring approximately 50 ms after an individual makes an error; the ERN is localized to anterior cingulate cortex and thought to reflect and associate with error monitoring processes (119). During an Eriksen flanker task, individuals with OUD exhibit faster reaction time to correct and incorrect trials than healthy controls, paired with smaller ERN amplitudes and faster latencies in frontocentral regions, suggestive of impairments related to impulsivity (103).

### Repetitive Transcranial Magnetic Stimulation (rTMS)

rTMS utilizes a handheld coil placed against the scalp, transmitting transient electric current to produce a changing magnetic field. This magnetic field can painlessly penetrate the skull and deliver a magnetic pulse to stimulate nerve cells in the brain. The TMS coil can be positioned to selectively target a region of the brain and excite or inhibit cortical neurons. rTMS studies are more common among other substance use disorders including alcohol, nicotine, and stimulants. However, one study employed rTMS within a sample of 20 men with OUD. This randomized, sham-controlled crossover study demonstrated that active but not sham 10 Hz rTMS over left dorsolateral PFC reduced craving induced by viewing videos of opioid use. Continued rTMS treatment for an additional 4 days further reduced cue-induced craving (120). These results are consistent with the *Preoccupation/Anticipation* stage of addiction wherein overactivation of frontal regions in response to cue-elicited craving drives preoccupation with drug-taking, suggesting that targeted rTMS stimulation of frontal regions may be a potential avenue for recovery in OUD.

### Deep Brain Stimulation (DBS)

In contrast to non-invasive rTMS, DBS is a invasive neuromodulation procedure administered via electrodes surgically implanted in subcortical brain areas. High frequency electrical stimulation is delivered to inhibit neural activity in targeted regions of the brain (121). DBS is used to treat movement disorders such as Parkinson's disease, and double-blind control trials show promise for its use in the treatment of refractory depression and obsessive compulsive disorder (122). Recently, DBS has been explored as an experimental treatment for patients with refractory substance use disorders, including OUD.

Among patients with OUD, DBS has been used to modulate activity in reward-network regions such as nucleus accumbens. Thus far, findings suggest that DBS is associated with partial to full remission and few side effects. For instance, within a small sample of chronic, treatment-resistant opioid users, DBS of the anterior limb of the internal capsule and nucleus accumbens resulted in prolonged sobriety greater than 2 years paired with reduced drug craving (123). Positron emission tomography scans also revealed increased glucose metabolism within bilateral IFG from pre- to post-DBS within these patients. Similarly, a case report demonstrated that an individual with a 5-year opioid use history underwent rapid detoxification and received DBS to bilateral nucleus accumbens for over 2 years. He subsequently maintained complete abstinence for the 6-year follow-up period after the electrode implantation surgery (121). Similarly, nucleus accumbens DBS in two chronic OUD patients resulted in decreased depression and anxiety paired with prolonged abstinence from opioids (124). However, an alternative case report of nucleus accumbens DBS stimulation in a man with 17 years of opioid use was unsuccessful in alleviating cravings 2 months post-DBS initiation. He relapsed eight times within the following 2 months and eventually overdosed within 5 months of DBS onset (122).

Abstinence following DBS treatment targeting reward-network regions is consistent with the *Binge/Intoxication* stage of addiction. DBS may reduce the reward response to drug use thereby interrupting the cycle that typically results in increased dopamine release and future drug use. While initial binge/intoxication may lead to incentive sensitization by weakening the brain's response to natural rewards in favor of drug rewards, use of DBS may interrupt the reward response, thereby reversing this process and allowing the brain to return to its initial preference for natural rewards (123, 125).

### Longitudinal Studies of Relapse and Treatment Outcome

Extant longitudinal neuroimaging studies of OUD combine imaging data with treatment to examine changes with treatment or baseline neural predictors of response. This research primarily concentrates on brain responses to drug cues, which within the context of abstinent individuals can be construed as appetitive and/or aversive. ERP results indicate that larger P300 amplitudes to opioid than pleasant images predicts greater opioid use frequency 6 months later (126), whereas lower frontal P300 amplitudes to non-drug distractors (127) and smaller ERN amplitudes during cognitive control (128) predict future treatment discontinuation. These findings point to executive function deficits within the *Preoccupation/Anticipation* stage that discount goals other than drug-seeking. Studies of fMRI prediction show that greater VS response (paired with higher self-reported craving) to opioid cues predicts relapse within 3 months (129), whereas higher medial PFC activation to opioid cues at baseline predicts more successful naloxone adherence (93) Additionally, functional connectivity fMRI studies demonstrate that although higher resting-state connectivity between ACC and medial PFC predicts relapse within 3 months (130), greater functional connectivity between INS, striatum, and ACC during a go/nogo task predicts successful 12-week substance use treatment (131). On the whole, these findings indicate that heightened salience of drug cues (particularly in striatal and frontal regions) forecasts difficulty maintaining sobriety, data congruent with the *Preoccupation/Anticipation* stage. Divergent task conditions across studies (cognitive control, resting-state, cue reactivity) may account for inconsistent findings; it would be helpful for future research to assess patterns of brain function across multiple paradigms to determine whether exaggerated or attenuated regions reflect global or context-dependent predictions.

Neuroimaging studies of OUD recorded at multiple timepoints demonstrate that naltrexone treatment: (1) decreases AMG and dorsal striatum signals while increasing medial PFC responses to opioid cues (132); (2) reduces VS and orbitofrontal responses to opioid cues as well as self- and clinician-reported withdrawal symptoms (133); and (3) increases VS activation to natural rewards (pictures of cute infants) (134). In contrast, a recent study shows that methadone maintenance treatment (>3 months) does not change frontocingulate mechanisms implicated in cognitive control during go/nogo task performance (135). These results convey that naltrexone shows promise in reducing appetitive (and perhaps aversive) salience of drug-related stimuli related to *Preoccupation/Anticipation* and *Negative Reinforcement* stages of addiction. Additional studies are warranted to replicate and extend these findings beyond naltrexone to buprenorphine and various therapy interventions. With respect to sMRI findings, OUD users completing 4 weeks of mindfulness-based treatment display improved striatum-INS and frontocingulate structural network strength than OUD users who received treatment as usual (136).

### Limitations and Gaps in Knowledge

Several gaps in the neuroimaging literature preclude development of accurate targets to identify and track treatment in OUD. First, inconsistent results are reported cross-sectionally for individuals with former OUD at various stages of recovery (from weeks to months) who also show wide variability in opioid use chronicity. Although testing interactions between drug use recency and chronicity may clarify inconsistent findings, this analysis has rarely been attempted (39). Longitudinal within-subjects designs provide increased statistical power to detect dynamic brain signal changes as a function of prolonged abstinence within each individual; however, few longitudinal neuroimaging studies tracking both brain and behavior change within OUD individuals exist, particularly accounting for both opioid use chronicity and recency. In addition, longitudinal designs can track changes in psychological symptoms related to negative mood states (e.g., depression and anxiety) that in conjunction with brain changes may distinguish OUD who relapse vs. those who are able to remain abstinent. Second, small sample sizes limit statistical power to detect potentially meaningful differences as a function of OUD status, and the majority of OUD studies are comprised of male participants [e.g., (50, 55, 57, 72, 74, 95, 101, 103, 107–109, 126)], limiting generalizability. Although more men use opioids than women, heroin use is increasing at a faster rate and prescription opioid use is decreasing at a slower rate among women than men, contributing significantly to the OUD crisis (137). In addition, research suggests that stress predicts opioid use in women but not men, pointing to the idea that *Negative Reinforcement* processes may be more crucial to target in women's recovery programs (138). Third, only a few OUD studies integrate neuroimaging methods with high temporal (EEG, ERPs) and spatial (sMRI, fMRI) resolution, limiting conclusions that can be drawn regarding precisely when and where brain processes change with abstinence. Longitudinal multimodal (EEG/ERP paired with sMRI, fMRI, and/or fNIRs) neuroimaging studies of OUD recovery are warranted to map temporal and spatial brain changes as a function of early vs. late stages of opiate abstinence and treatment outcome, while mapping changes in individual differences in psychological symptoms [e.g., depression and anxiety; (12, 13)] and co-use of other substances (e.g., alcohol, nicotine) (139). Lastly, despite the fact that processing during the *Negative Reinforcement* stage of addiction is theorized to drive users to relapse (140), few neuroimaging studies of OUD have evaluated how aversive or stressful stimuli, alone or in conjunction with opioid cues, transform brain circuitry to hijack intended abstinence efforts and drive relentless capitulation to drug use despite increasingly dire consequences. The following sections highlight two promising avenues of research that can evaluate aversive sensitization in individuals with OUD.

### Operant Conditioning and Interoception

Interoception, the perception and awareness of bodily signals, is thought to be dysregulated as a function of addiction, contributing to drug craving and urges (26, 141–144), but only two studies have examined interoceptive processing in OUD, demonstrating impaired interoceptive awareness as measured by heartbeat tracking accuracy (145), and greater stress-related physiological arousal and craving in response to paired pain-opioid stimuli as a function of pain-driven opioid misuse (146). However, no neuroimaging studies have probed the integrity of brain circuitry implicated in aversive interoceptive processing in OUD. Work by our research team demonstrates that, within the context of an aversive interoceptive manipulation (inspiratory breathing load), stimulant use disorder is characterized by exaggerated trait anxiety paired with attenuated striatum, INS, IFG, and ACC responses during decision-making (147–149). These findings point to increased arousal mismatched with blunted processing of bodily signals in the absence of drug-related stimuli, a pattern that could translate into impaired awareness of or attention to negative consequences during real-world decision-making consistent with the *Preoccupation/Anticipation* and *Negative Reinforcement* stages of addiction. Future studies could attempt to replicate this brain-based pattern of blunted aversive interoceptive processing in OUD and then extend this work by pairing aversive interoception with the presence vs. absence of drug cues to test the role of opioids in aversive sensitization.

### Classical Conditioning and Extinction

Fear conditioning is a process where individuals learn which cues are associated with aversive outcomes (shocks, sounds, odors). With repetitive cue-outcome pairings, the cue alone can trigger the same response as the aversive outcome (conditioned fear). A recent meta-analysis demonstrates that fear-conditioned cues consistently elicit greater INS, striatum, and frontocingulate responses than unconditioned cues (150). Heightened AMG signaling for fear-conditioned cues is present across several studies, but may vary across tasks as a function of stimulus duration, predictability, and presentation modality [e.g., (151–156)]. Exaggerated physiological arousal during fear conditioning is specifically associated with AMG-INS signaling and connectivity (157, 158). Fear extinction, in contrast to conditioning, is the process wherein individuals learn to dissociate cues from their previously paired aversive outcomes, involving INS and ACC across studies (159) as well as AMG, particularly within early extinction (153, 160, 161). No studies have examined whether brain mechanisms of classical conditioning and extinction are intact in OUD within the context of aversive stimuli, but given behavioral impairments in decision-making as a function of punishment in OUD (64, 79–82), it is possible that associative learning and unlearning involving negative stimuli is disrupted in opioid users. Future research could identify whether brain circuitry impairments to fear-conditioned and extinguished-stimuli characterizes OUD in the presence vs. absence of drug cues.

## Summary and Conclusions

Delineating neuroimaging targets for recovery from OUD is a difficult task, given that the majority of studies investigating abstinence are cross-sectional, comprised of opiate users with heterogeneous patterns of use chronicity and recency that may complicate results. In particular, methadone maintained individuals with OUD show brain impairments that are more similar to active illicit opioid users than individuals abstinent from opioids altogether. However, longitudinal studies show some promise that other treatments (e.g., rTMS, DBS, and naltrexone) or prolonged abstinence can change brain signals implicated in *Negative Reinforcement* and *Preoccupation/Anticipation* to reduce salience of drug cues, which may attenuate craving and anguish driving individuals to resume opioid use. The pairing of cue-reactive stimuli with established paradigms targeting cognitive control (e.g., flanker, go/nogo, stop signal) and/or emotion regulation [cognitive reappraisal of negative stimuli; e.g., (162)] may be beneficial for tracking the degree of brain resources that continue to be captured by drug cues over the course of recovery. Many more longitudinal investigations, particularly with males and females and within the context of aversive or stress-related stimuli, are warranted to develop individual-difference prediction models of recovery in OUD.

## Author Contributions

JS wrote the first draft of the manuscript with AM's input and created the figures. AM, RA, and JB provided revisions to further manuscript drafts. AM formatted the manuscript for publication.

### Conflict of Interest Statement

The authors declare that the research was conducted in the absence of any commercial or financial relationships that could be construed as a potential conflict of interest.
